# Determination of Reaction Enthalpies of Synthesizing β-Li_3_PS_4_ in Tetrahydrofuran

**DOI:** 10.1021/acsomega.3c00603

**Published:** 2023-04-06

**Authors:** Aurelia Gries, Frederieke Langer, Julian Schwenzel, Matthias Busse

**Affiliations:** † Fraunhofer Institute for Manufacturing Technology and Advanced Materials IFAM, Lilienthalplatz 1, 38108 Braunschweig, Germany; ‡ Fraunhofer Institute for Manufacturing Technology and Advanced Materials IFAM, Wiener Straße 12, 28359 Bremen, Germany

## Abstract

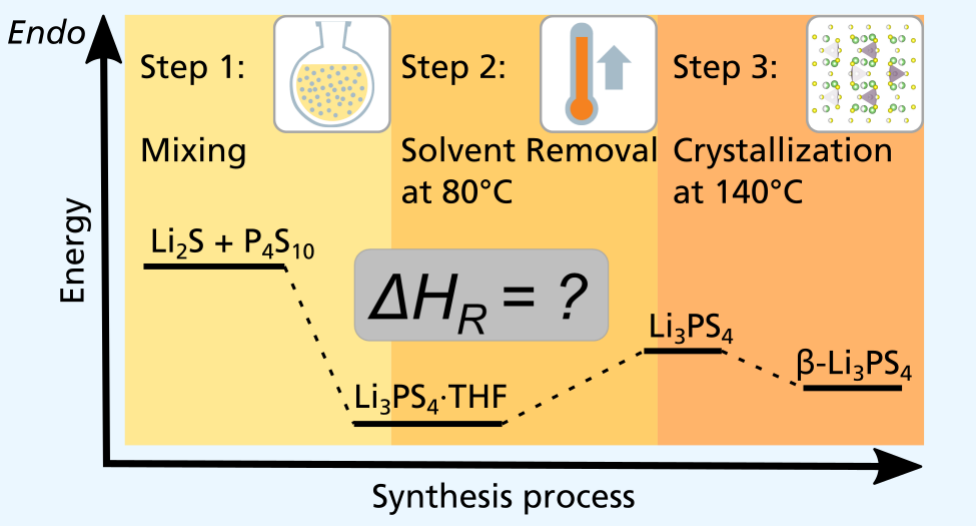

While some promising materials for all-solid-state batteries are already extensively investigated in a lab scale, the transferability to mass production is still a limiting factor. β-lithium thiophosphate (β-Li_3_PS_4_) has good ionic conductivity and can be synthesized wet-chemically, which opens up the possibility for scale-up. For safe upscaling, the enthalpies of the synthesis steps need to be examined in order to handle exothermic and endothermic processes. Here, the reaction enthalpies of the wet-chemical synthesis of β-Li_3_PS_4_ in tetrahydrofuran (THF) are determined. The synthesis routine is established in a lab scale, and the synthesis success is confirmed via X-ray diffraction (XRD) and electrochemical impedance spectroscopy (EIS). The reaction of the educts in THF is investigated using a reaction calorimeter and shows a strongly exothermic process. The subsequent processes are examined using differential scanning calorimetry with thermogravimetric analysis and show a strong endothermic process during solvent removal and a slightly exothermic process during crystallization.

## 1 Introduction

All-solid-state batteries are considered the next generation in battery technology due to increased energy and power density as well as freedom of design, e.g., constructing batteries in bipolar stacks.^1,2^ Solid-state electrolytes offer advantages over conventional liquid electrolytes, such as greater thermal safety, because of high melting points and higher mechanical strength, which is believed to suppress the growth of lithium dendrites and, therefore, opens up the possibility of using a lithium metal anode. One promising material group is sulfides,^3−5^ of which crystalline β-Li_3_PS_4_ gathered much attention over the last few years due to its good ionic conductivity (∼1.6 × 10^–4^ S/cm at 25 °C^6^) and the opportunity of using a wet-chemical synthesis route.

The wet-chemical synthesis offers advantages such as shorter reaction times, lower temperatures, and tailoring of particle sizes^7^ as well as facilitated scaling-up of the production compared to high-temperature solid-state reactions and the mechanical ball-milling route.^8−11^ For safe upscaling, it is important to examine the enthalpies of the synthesis steps in order to handle exothermic and endothermic processes. While uncontrolled heat or gas development of exothermic reactions can cause damage to the reaction equipment and severe safety issues, excessive heat dissipation can slow down the reaction kinetics. For endothermic reactions, it is necessary to provide the reagents with enough energy to facilitate and accelerate the reaction while considering the thermal stability of the reactants. However, today, there is no report of the heat development of this synthesis, which is important for scaling up the synthesis safely.

The wet-chemical synthesis route was developed by Liu et al.^6^ In short, the synthesis consists of mixing the educts lithium sulfide (Li_2_S) and phosphorus pentasulfide (P_4_S_10_) in a stoichiometry of 3:1 in anhydrous tetrahydrofuran (THF) at room temperature overnight. Afterward, the intermediate Li_3_PS_4_·3THF is obtained by centrifugation. Heat treatment at 80 °C under vacuum is used for the removal of THF, which results in amorphous Li_3_PS_4_. By heating at 140 °C under vacuum, β-Li_3_PS_4_ is obtained. While the synthesis is already examined regarding the obtained particle size,^7^ crystallization temperature and duration,^12,13^ different solvents,^9,14−16^ and reaction mechanism,^17−19^ there are no reports on thermal parameters.

In this work, the reaction enthalpies of synthesizing β-Li_3_PS_4_ in THF are determined by calorimetry. First, the synthesis in a lab scale is established and the intermediates and product are used for verification of synthesis success and further experiments. Based on the synthesis routine of Liu et al.,^6^ the synthesis is performed by mixing the educts in THF, separating the intermediate by centrifugation, and crystallizing to β-Li_3_PS_4_ by heat treatment.

To scale up the synthesis, the energy balances are important to know. Therefore, we focus on the reaction enthalpies of the chemical steps:

Chemical steps:


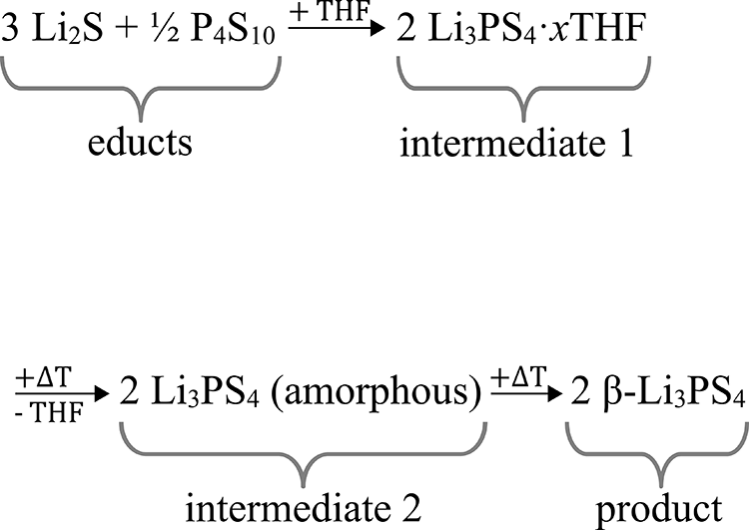

(1) The educts Li_2_S and P_4_S_10_ react in THF to form solid Li_3_PS_4_·*x*THF (intermediate 1). The stoichiometric share (*x*) of THF is calculated from TGA results.
(2) THF in intermediate 1 is removed by heat treatment at 80 °C under vacuum resulting in amorphous Li_3_PS_4_ (intermediate 2).
(3) Through further heat treatment at 140 °C under vacuum, intermediate 2 crystallizes to β-Li_3_PS_4_ (product).

Examination of the reaction mechanism showed that the first step contains two substeps: (a) The reaction between Li_2_S and P_2_S_5_ in a ratio of 1:1 and (b) the further reaction with the residual Li_2_S.^19^ In order to determine the reaction enthalpy of the first step and the first substep, a reaction calorimeter is used while differential scanning calorimetry (DSC) is used to determine the enthalpies of the two heating steps. The aim of these experiments is to describe the reaction enthalpies of the wet-chemical synthesis of β-Li_3_PS_4_ in THF. This contributes to the development of a scaled-up synthesis route for β-Li_3_PS_4_.

## 2 Methods

### 2.1 Theoretical Considerations

Estimating the overall reaction enthalpy according to Hess’s law^20^ is a common approach to calculate reaction enthalpies. Here, the total enthalpy of a reaction Δ*H*_R_ is assumed independent of the reaction steps and, therefore, summing up the standard enthalpies of formation Δ*H*^0^ of educts and products results in the same value as summing up the reaction enthalpies of all separate steps of the reaction. While for Li_2_S, a well-defined standard enthalpy of formation (Δ*H*^0^(Li_2_S) = −441.4 kJ/mol^21^) is reported, a certain degree of uncertainty arises from the state of phosphorus pentasulfide. In general, phosphorus pentasulfide occurs as P_4_S_10_ because P_2_S_5_ is the more reactive and metastable state.^22^ Furthermore, Thamm et al.^23^ reported that commercial phosphorus pentasulfide contains about 70 mol % P_4_S_9_ and 30 mol % P_4_S_10_. Hence, the value for phosphorus pentasulfide is somewhere between Δ*H*^0^(P_4_S_10_) = −308.9 kJ/mol^24^ and Δ*H*^0^(P_4_S_9_) = −292.1 kJ/mol.^24^ Finally, for the standard enthalpy of formation of β-Li_3_PS_4_, there is no experimental data. Calculated values were reported by Lepley et al.^25^ It is found that the standard enthalpies of formation for different idealized structures of β-Li_3_PS_4_ vary between Δ*H*^0^(β-Li_3_PS_4_) = −798.9 and −789.25 kJ/mol. The structure with the highest negative value was found to be the most stable one.

For the reaction investigated in the present work, the reaction enthalpy is calculated as follows
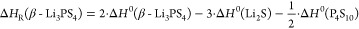
Due to the uncertainties discussed above, the total reaction enthalpy is calculated between Δ*H*_R_(β-Li_3_PS_4_) = −349.7 and −279.8 J/g.

### 2.2 Experimental Section

#### 2.2.1 Lab Scale Synthesis

Briefly, 1.91 g of Li_2_S (Alfa Aesar, 99.9%) and 3.09 g of P_4_S_10_ (Sigma-Adrich, 99%) were mixed in 25 mL of THF (VWR Chemicals, anhydrous, max. 30 ppm H_2_O) and stirred with a magnetic stirrer for 24 h in an argon-filled glovebox. Afterward, the excess solvent was separated by centrifuging and decanting. The received solid intermediate 1 was treated with two heating steps: First, the THF of intermediate 1 was removed by heating at 80 °C for 4 h under vacuum forming intermediate 2. Subsequently, the temperature was increased to 140 °C and the sample was kept for 12 h under vacuum for crystallization of intermediate 2 to β-Li_3_PS_4_. A fraction of intermediate 1 was stored to perform DSC.

#### 2.2.2 Characterization of Intermediates and the Synthesis Product β-Li_3_PS_4_

##### 2.2.2.1 XRD

The phase purity and crystal structure of synthesis intermediates and the final product were determined by XRD using an inert sample holder in an X-ray diffractometer (Mini Flex 600, Rigaku). The samples were prepared in an argon-filled glovebox. The instrument is equipped with a Cu(Kα)-source and a stepwise scan was carried out from 5 to 60° 2θ with a step size of 0.03° 2θ.

##### 2.2.2.2 Electrochemical Impedance Spectroscopy

Pellets of 150 mg of β-Li_3_PS_4_ were pressed in a hydraulic press with 45 bar for 5 min at room ambient temperature (RT). The diameter was 10 mm and the height was about 1 mm. As symmetric blocking electrodes, carbon-coated aluminum foil was used. The impedance measurement was performed by a Gamry Interface 1010E between 1 MHz and 1 Hz with an amplitude of 10 mV in KP solid cells (Hohsen) with a pressure of 15 MPa. The temperature was varied between −40 and 60 °C. The measurement data was processed with RelaxIS 3 (RHD instruments).

#### 2.2.3 Thermal Analysis

##### 2.2.3.1 Calorimetry (Chemical Step 1)

**Figure 1:**
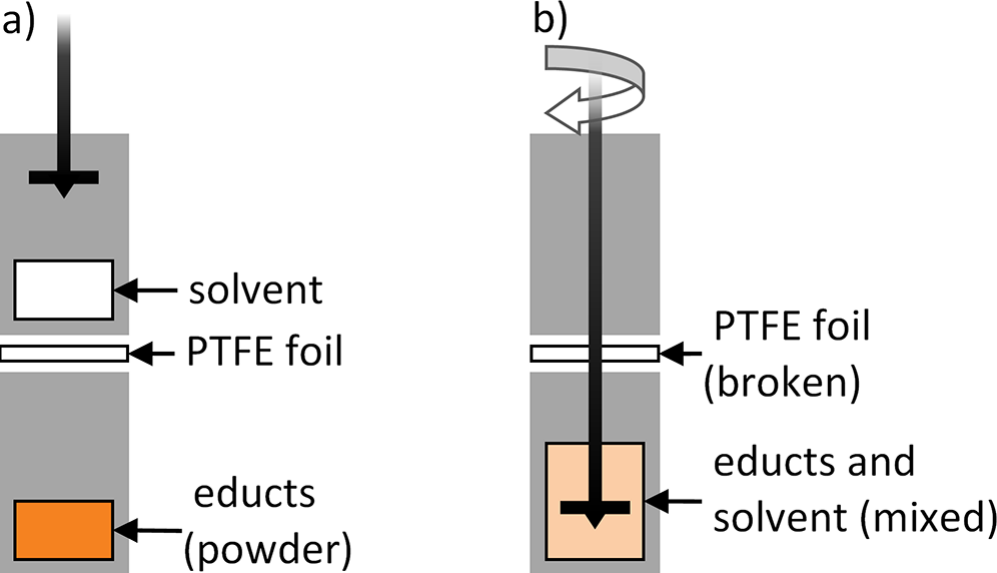
Schematic representation of the mixing vessel for a Calvet C80. (a) Preparation and (b) mixing.

For the examination of the first chemical step, a Calvet C80 calorimeter (Setaram) was used. A mixing vessel with a PTFE membrane allows for examining the process under an inert atmosphere. A schematic representation is shown in Figure 1. For sample preparation, the ratios of educts and solvent were scaled down to 100 mg of the product mass. Therefore, 38 mg of Li_2_S and 62 mg of P_4_S_10_ were given in the bottom chamber of the test tube, which was then sealed with PTFE foil. The upper chamber was filled with 450 mg of THF (Figure 1a). A reference tube was also filled with argon and THF. The sample named “X/Y” indicates the ingredients of the sample tube vs ingredients of the reference tube. “LPS” serves as the abbreviation of the mixture of Li_2_S and P_4_S_10_. To start the reaction LPS + THF/THF, the stirrers of both tubes were pushed down to pierce the foil and let the solvent flow into the bottom chamber (Figure 1b). The reaction was performed at 30 °C, and the stirrers were rotated at 60 rpm. The measurement was conducted thrice for reproducibility.

Furthermore, the reaction enthalpies were determined for the sole educts:

(1) THF/THF: 450 mg of THF in a test tube and 450 mg of THF in a reference tube.
(2) Li_2_S + THF/THF: 38 mg of Li_2_S and 450 mg of THF in a test tube and 450 mg of THF in a reference tube.
(3) P_4_S_10_ + THF/THF: 62 of mg P_4_S_10_ and 450 mg of THF in a test tube and 450 mg of THF in a reference tube. For measuring the enthalpy of the first substep, containing the reaction of the educts in a Li_2_S/P_2_S_5_ ratio of 1:1, the following samples were prepared:
(4) LPS(1:1) + THF/THF: 13 mg of Li_2_S, 62 mg of P_4_S_10_, and 450 mg of THF in a test tube and 450 mg of THF in a reference tube.

##### 2.2.3.2 DSC/TGA (Chemical Steps 2 and 3)

Differential scanning calorimetry (DSC) with thermogravimetric analysis (TG) was performed with a simultaneous thermal analyzer (STA 449 F3 Jupiter, Netzsch). The synthesized intermediate Li_3_PS_4_·*x*THF was heated from 35 to 160 °C with 1 K/min under argon flow in a sealed Al pan. Immediately before measurement, the Al pan was pricked to allow escaping of evolving gases that were expected to form at temperatures above 66 °C (boiling point of THF) from intermediate 1. The high mass of argon and the argon flow ensure that the contact of the sample with air is prevented and, therefore, the integrity of the inert measurement.

## 3 Results and Discussion

### 3.1 Lab Scale Synthesis

After adding the powdery educts to THF, the temperature increases significantly from 27 °C to about 65 °C implying the start of an exothermic reaction. The temperature reaches a maximum after about 3 min and then decreases exponentially over the following hours. The successful synthesis of intermediates and the final product is verified by analyzing the crystal structure after each synthesis step.

**Figure 2:**
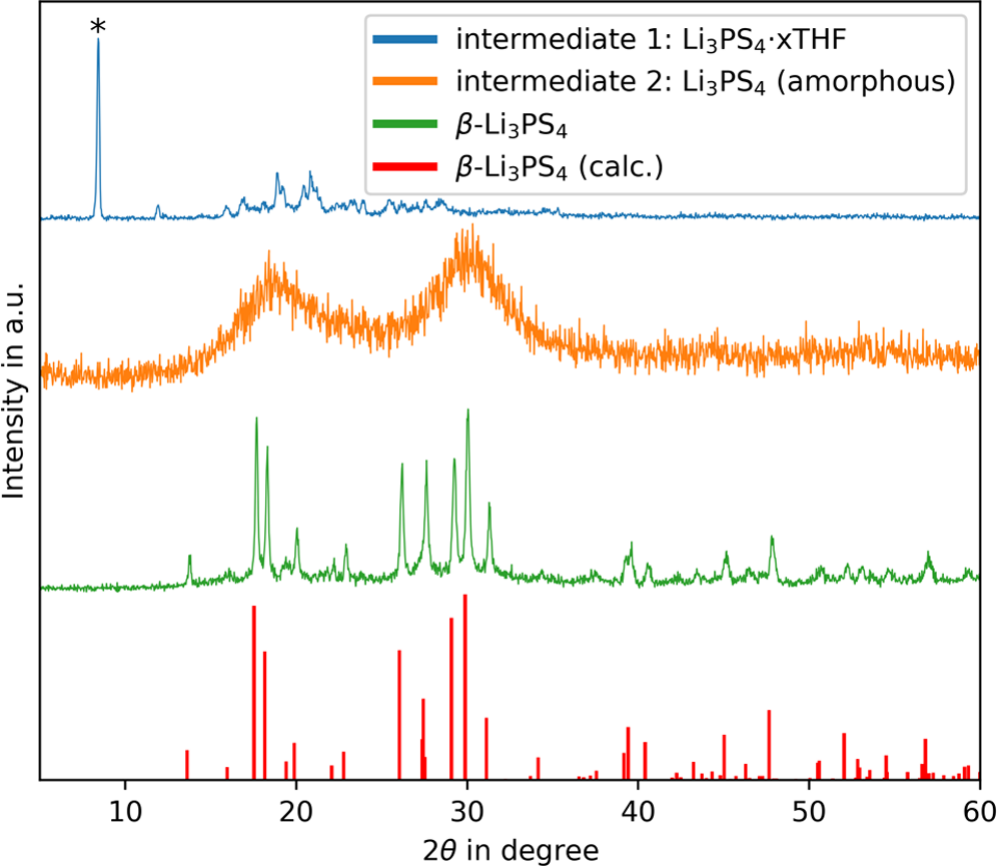
Diffractograms of intermediates and the synthesis product β-Li_3_PS_4_.

The obtained intermediates (Li_3_PS_4_·*x*THF (intermediate 1), amorphous Li_3_PS_4_ (intermediate 2)), and the product β-Li_3_PS_4_ are examined via XRD (Figure 2). Intermediate 1 is collected after mixing and is represented by the blue line. The pattern shows the typical strong reflection of Li_3_PS_4_·*x*THF at 2θ ≈ 8.4°, indicated with an asterisk, and some other smaller reflections, being in good agreement with previous reports.^6,26^ The orange line shows the XRD pattern of intermediate 2, collected after heat treatment at 80 °C, which is amorphous Li_3_PS_4_. This is evidenced by the broad amorphous shoulders at 2θ ≈ 18° and 2θ ≈ 30° and the absence of sharp reflections. The XRD pattern of the final product β-Li_3_PS_4_, after further heat treatment at 140 °C, is plotted by the green line. All reflections can be assigned to β-Li_3_PS_4_ by matching the pattern with structural data calculated from Stöffler et al.^27^ (red lines). Since no further reflections are observed, the phase purity of the final product is assumed. Overall, the XRD patterns are in good agreement with those of Liu et al’s.^6^ and other previous reports and, therefore, the synthesis routine is suitable to obtain β-Li_3_PS_4_ and the intermediates.

**Figure 3:**
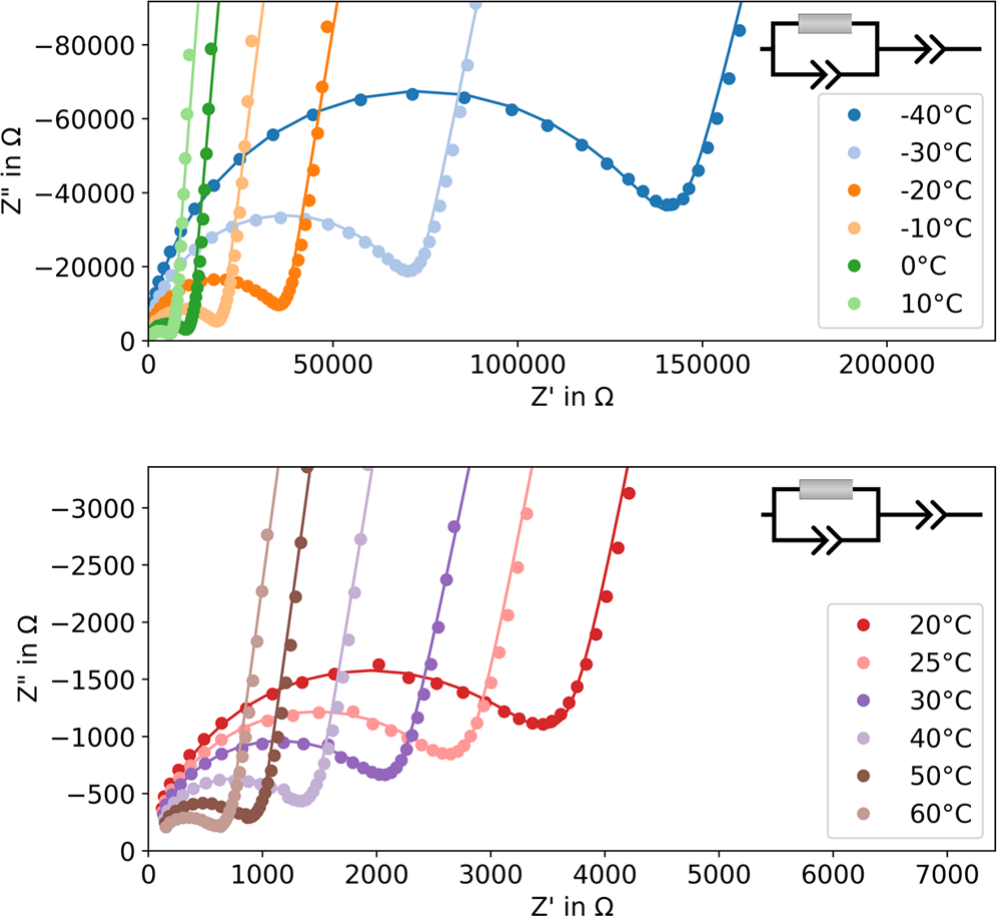
Exemplary Nyquist plots of experimental (points) and fitted (line) temperature-dependent electrochemical impedance measurements for the synthesis product β-Li_3_PS_4_. The inset depicts the equivalent circuit used for fitting.

**Figure 4:**
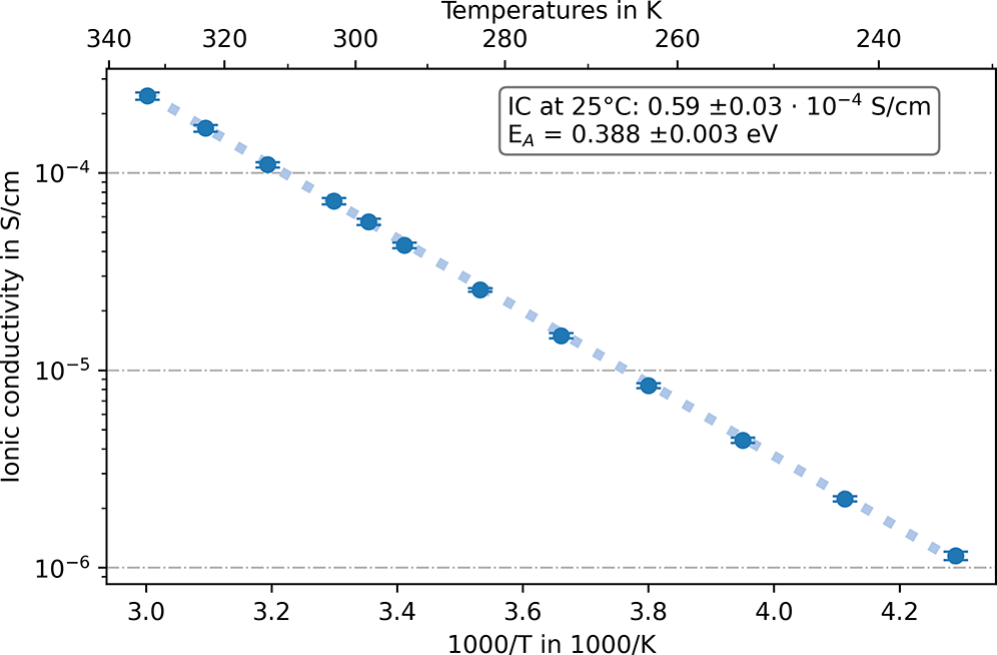
Temperature-dependent ionic conductivity of the synthesis product β-Li_3_PS_4_ shown in an Arrhenius plot.

The impedance measurements show the typical response of an ionic conductor placed between two blocking electrodes. Therefore, an established equivalent circuit consisting of a resistor with a constant phase element in parallel and a further constant phase element in a row is used for fitting the data. Exemplary Nyquist plots showing the measured and fitted data are shown in Figure 3. The ionic conductivity is calculated from the obtained resistances using the height and diameter of the pellets. In Figure 4, the temperature-dependent ionic conductivity of three pellets of the synthesis product β-Li_3_PS_4_ is shown. At room temperature, the ionic conductivity is 0.59 ± 0.03 × 10^–4^ S/cm with a low deviation between the pellets. This is slightly lower than the values found in the literature^6,28^ and may be due to the lower density of the pellets in comparison to other reports. Here, 79 ± 1% compared to the bulk material is achieved by uniaxial cold pressing, while Liu et al.^6^ reported 95%. The calculated activation energy is 0.388 ± 0.003 eV, which is also similar to the value reported by Liu et al.^6^ (0.356 eV).

Using XRD and EIS measurements, the successful synthesis of intermediates and the final product β-Li_3_PS_4_ in the lab scale is verified and, therefore, the determination of the enthalpies of the chemical steps can be performed scaled down in the reaction calorimeter and the simultaneous thermal analyzer.

### 3.2 Thermal Analysis

#### 3.2.1 Calorimetry of the Mixing Step

The mixing of synthesis educts is performed in a calorimeter and the heat flow is recorded continuously. The enthalpies are calculated by determining the area between the baseline and the measured heat flow. For the sole educts, normalized to the synthesis of 1 g of β-Li_3_PS_4_, the following values are obtained
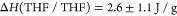

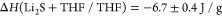
and
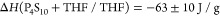
For the blank measurement THF/THF, a low positive value is measured. Compared to the order of magnitude of the experiments performed subsequently, this value is rather small. While the value of Li_2_S + THF/THF is in the same order of magnitude as that of the blank measurement THF/THF, the value for P_4_S_10_ + THF/THF is one magnitude higher than that for the previous experiments, indicating a chemical process. Although both educts, Li_2_S and P_4_S_10_, are described as hardly soluble in THF,^29^ the exothermic reaction after adding THF to P_4_S_10_ indicates a chemical process, most likely a partial dissolution of P_4_S_10_ in THF. This is in agreement with other reports proposing that the dissolution of P_4_S_10_ is the initial step of the reaction.^9,18^

**Figure 5:**
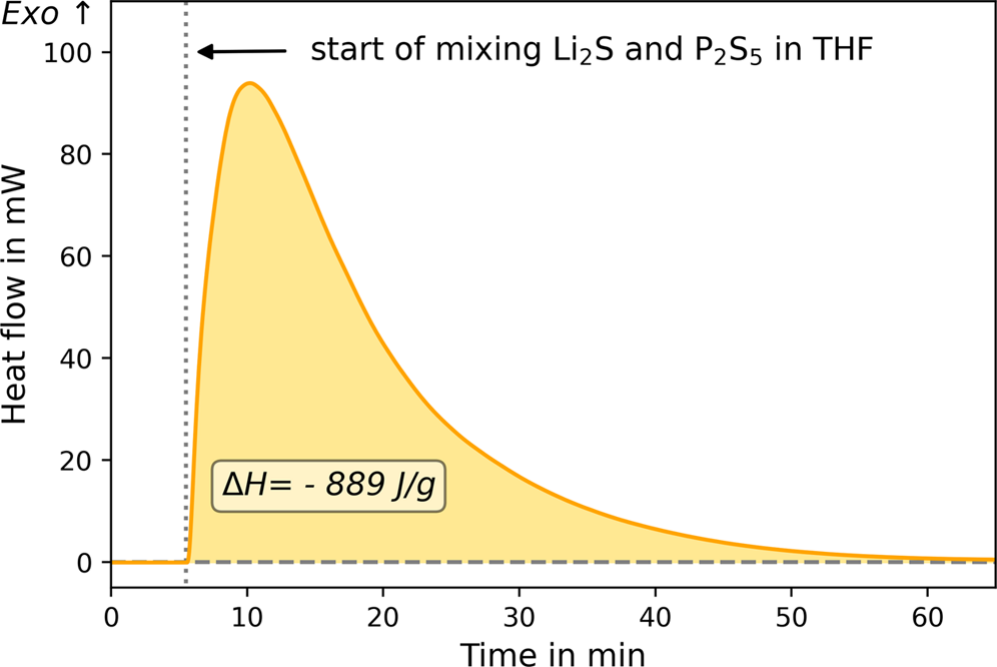
Heat flow of the reaction of lithium sulfide and phosphorus pentasulfide in THF (chemical step 1).

In contrast, the heat evolution is more pronounced, when THF is added to the mixture of both educts (LPS + THF/THF). The obtained signal is shown in Figure 5. As soon as the membrane between the powders and solvent is pierced (indicated in Figure 5 by the dotted line), letting the solvent reach the bottom chamber, a strong heat flow signal is detected. In the beginning, an exothermal heat flow increases strongly, with a maximum of 94 mW after 5 min of mixing. Afterward, the heat flow decreases and the end of the experiment is determined after 60 min when the heat flow reaches values equal to values prior to mixing. The baseline is depicted as the dashed line. For the reaction, a normalized reaction enthalpy of Δ*H* (LPS + THF/THF) = −889 ± 12 J/g is calculated.

Furthermore, the reaction enthalpy of a mixture of the educts in a Li_2_S/P_2_S_5_ ratio of 1:1 is examined, which was found to be the first substep of the reaction between the educts.^19^ Here also, a strongly exothermic reaction is detected, resulting in a normalized reaction enthalpy of Δ*H* (LPS(1:1) + THF/THF) = −701 ± 18 J/g. The value for the 1:1 ratio is smaller than for the 3:1 ratio examined before, indicating that the first substep containing the reaction between the educts in a Li_2_S/P_2_S_5_ ratio of 1:1 is not the only source of the reaction enthalpy measured for (LPS + THF/THF) and that the second reaction substep during the mixing is exothermic as well.

Accordingly, the wet-chemical synthesis route contains a highly exothermic reaction step, which is caused by all reactants. The finding of heat dissipation is essential for the design of a safe setup for scaling up the synthesis.

#### 3.2.2 Solvent Removal and Crystallization (DSC/TG)

**Figure 6:**
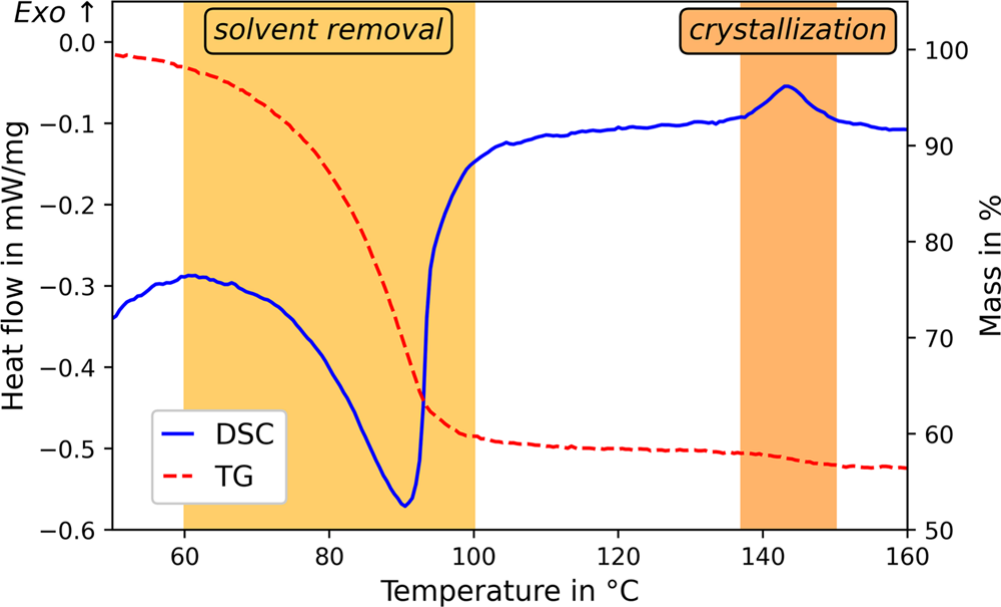
DSC and TG of the synthesis of the intermediate Li_3_PS_4_·*x*THF (chemical steps 2 and 3).

The thermal treatment steps for solvent removal and crystallization are investigated using simultaneous thermal analysis. Figure 6 shows the DSC signal as a blue line, while the red dashed line represents the mass loss. The DSC graph depicts two distinct peaks. The first peak shows an endothermic process between 60 and 100 °C with a peak at 90 °C, which coincides with a major mass loss in the TG graph. Since the boiling point of THF is at 66 °C, the thermal event is in agreement with the boiling and evaporation of THF. Hence, the DSC signal is attributed to the removal of THF from Li_3_PS_4_·*x*THF (chemical step 2). The necessary amount of energy for the dissociation of THF is calculated Δ*H* (step 2) = 748 ± 7 J/g. In addition, the DSC measurement shows an exothermic peak with an onset temperature of 140 °C, which is Δ*H* (step 3) = −45.0 ± 0.1 J/g. Since this DSC peak does not coincide with a major mass loss in the TG graph, it indicates a phase transition process. Hence, it is attributed to the crystallization of intermediate 2 to β-Li_3_PS_4_. The assignment of the peaks to the chemical processes is supported by the XRD measurement discussed above.

Nearly all mass loss (∼42%) appears during the removal of THF, while crystallization removed the last residuals of trapped THF (∼2%). The residual mass at 160 °C is 56.0 ± 0.7%. Derived from the total mass loss of 44%, the stoichiometric share of THF in intermediate 1 is *x* = 2, and the chemical formula results in Li_3_PS_4_·2THF. In Liu et al.,^6^ a stoichiometry of 1:3 is described, whereas Marchini et al.^30^ reported 1:2, being in good agreement with our findings.

**Figure 7:**
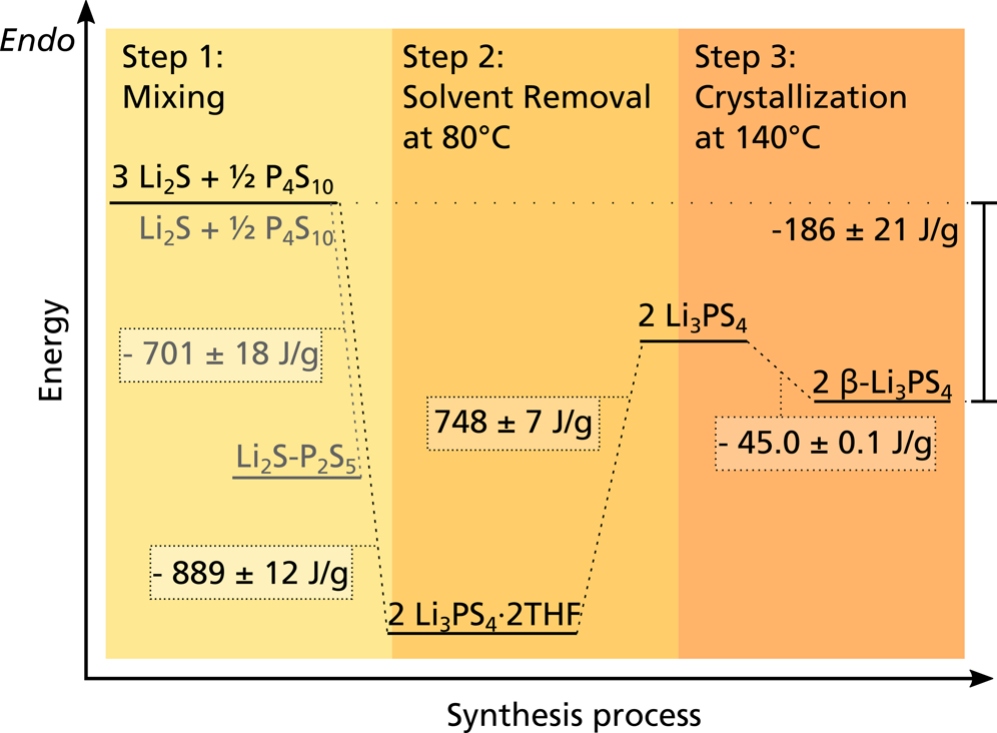
Energy diagram showing the reaction enthalpies for synthesizing β-Li_3_PS_4_ in THF.

The thermal analysis provides the values needed to develop the energy diagram for the synthesis route shown in Figure 7. The first step is strongly exothermic, including the intermediate step for the 1:1 ratio, which is shown in gray. While the second step has a high energy demand, the third step releases a small amount of energy. The overall reaction enthalpy results in Δ*H*_R_(3Li_2_S + ^1^/_2_P_4_S_10_ → 2β-Li_3_PS_4_) = −186 J/g.

The reaction enthalpy calculated in Section 2.1 is between Δ*H*_R_(β-Li_3_PS_4_) = −349.7 and −279.8 J/g, which is in the same order of magnitude as the experimental data. The upper and the lower limit of this estimation show a wide range, but still, the experimental value is significantly different. This may rise from several influencing factors. As previously discussed, the uncertainty of the theoretical values used in Hess’s law contributes to deviations. Furthermore, the values of the standard enthalpy of formation of β-Li_3_PS_4_ are simulated values, which typically show deviations from experimental data, as, for example, reported by Lepley et al.^25^ Furthermore, Hess’s law is used under the assumption that the reaction enthalpy is independent of the reaction steps. It is likely that the enthalpies of the practical synthesis pathway differ from the theoretically predicted value due to processes, which are not considered in Hess’s law. An example of mechanical effects is the energy contribution by stirring due to the higher friction caused by the movement of the solid in the first step. Also, practical drying and crystallization contributions due to effects like surface adsorption are not represented.

Using Pair Distribution Function calculations is another option to estimate enthalpies. A formation enthalpy of −1.78 eV/2Li_3_PS_4_ to form Li_3_PS_4_ from lithium sulfide and phosphorus pentasulfide was predicted by Lim et al.^31^ This equals an enthalpy of Δ*H*(Li_3_PS_4_) = −477 J/g, but it is not clear which state of the reaction was chosen as the last step. The last step is described as the precipitation of crystalline Li_3_PS_4_ from solution. Therefore, the value is probably comparable with the first chemical reaction step of our process, which is Δ*H*(step 1) = −889 ± 12 J/g. Under this assumption, the absence of a solvent as in Li_3_PS_4_·2THF might explain the divergence between calculated and measured values.

Regarding the transfer of the synthesis to larger batch sizes, the strongly exothermic behavior of the mixing step is crucial to quantify for synthesizing larger amounts of β-Li_3_PS_4_ safely. For the small batch size, the surface-to-volume ratio is high. Therefore, a large amount of heat can dissipate over the surface. With increasing batch size, the surface-to-volume ratio decreases, potentially below the critical value necessary to dissipate the heat in order to avoid boiling the suspension. While there is a multitude of aspects to be considered during scaling, the following four considerations with regard to heat management are made: First, the synthesis could be cooled. However, cooling the reaction vessel requires balancing the heat removal without strongly inhibiting reaction kinetics. Second, stepwise addition of the solid educts to the reaction vessel slows down the heat development. Due to the majority of heat occurring during the reaction of Li_2_S/P_2_S_5_ = 1:1, it is an option to first add the educts in a ratio of 1:1 and add the additional Li_2_S later. Still, this could prolong the synthesis duration and complicate the material flow. Third, a lower solid concentration could be used, but this is likely to influence the particle size,^7^ as well as the stoichiometry due to the dissolution of P_2_S_5_ in the increased solvent volume. Fourth, solvents with different thermal properties like higher heat capacity and higher boiling point, e.g., ethyl acetate^32^ or ethyl propionate,^33^ could be used. A higher heat capacity leads to the intake of a higher amount of heat at the same solvent amount and, therefore, the temperature increase will be smaller. Furthermore, a higher boiling point opens up a larger working range. However then, the usage of another solvent might also change the reaction enthalpies, and the impact of the solvent on the later process steps, especially drying, has to be considered. Just like the change in the solvent fraction, another solvent can also influence the particle morphology.^33^

## 4 Conclusions

In this work, the reaction enthalpies of the wet-chemical synthesis of β-Li_3_PS_4_ are determined. In this context, the success of the synthesis routine for synthesizing β-Li_3_PS_4_ and its intermediates is confirmed by XRD and EIS. The reaction enthalpies for the chemical steps (mixing, solvent removal, and crystallization) are investigated for each step. The exothermic value of the reaction of lithium sulfide and phosphorus pentasulfide in tetrahydrofuran is Δ*H*(step 1) = −889 ± 12 J/g. Furthermore, the enthalpies for the other two steps are Δ*H*(step 2) = 748 ± 7 J/g and Δ*H*(step 3) = −45.0 ± 0.1 J/g. Therefore, the overall reaction enthalpy results in Δ*H*_R_(Li_2_S + P_4_S_10_ → β-Li_3_PS_4_) = −186 J/g. In addition, the dissolution of P_4_S_10_ in THF shows a small exothermic value, which supports the hypothesis of this being the initial step of the reaction mechanism.

To scale up the wet-chemical synthesis route, the reaction enthalpies are needed in order to handle exothermic and endothermic processes. Especially, knowledge about the reaction enthalpy of the mixing step is essential to scale up the process safely. For the investigated batch size and solvent amount, the obtained maximum temperature is scarcely below the boiling point of the chosen solvent. Therefore, heat dissipation has to be considered for larger batch sizes.
